# Machine learning to establish three sphingolipid metabolism genes signature to characterize the immune landscape and prognosis of patients with gastric cancer

**DOI:** 10.1186/s12864-024-10243-z

**Published:** 2024-03-28

**Authors:** Jianing Yan, Xuan Yu, Qier Li, Min Miao, Yongfu Shao

**Affiliations:** Department of Gastroenterology, The First Affiliated Hospital of Ningbo University, 315020 Ningbo, China

**Keywords:** Sphingolipid metabolism, Multi omics, Gastric cancer, Prognosis, Nomogram, Immune infiltration

## Abstract

**Background:**

Gastric cancer (GC) is one of the most common malignant tumors worldwide. Nevertheless, GC still lacks effective diagnosed and monitoring method and treating targets. This study used multi omics data to explore novel biomarkers and immune therapy targets around sphingolipids metabolism genes (SMGs).

**Method:**

LASSO regression analysis was performed to filter prognostic and differently expression SMGs among TCGA and GTEx data. Risk score model and Kaplan-Meier were built to validate the prognostic SMG signature and prognostic nomogram was further constructed. The biological functions of SMG signature were annotated via multi omics. The heterogeneity landscape of immune microenvironment in GC was explored. qRT-PCR was performed to validate the expression level of SMG signature. Competing endogenous RNA regulatory network was established to explore the molecular regulatory mechanisms.

**Result:**

3-SMGs prognostic signature (GLA, LAMC1, TRAF2) and related nomogram were constructed combing several clinical characterizes. The expression difference and diagnostic value were validated by PCR data. Multi omics data reveals 3-SMG signature affects cell cycle and death via several signaling pathways to regulate GC progression. Overexpression of 3-SMG signature influenced various immune cell infiltration in GC microenvironment. RBP-SMGs-miRNA-mRNAs/lncRNAs regulatory network was built to annotate regulatory system.

**Conclusion:**

Upregulated 3-SMGs signature are excellent predictive diagnosed and prognostic biomarkers, providing a new perspective for future GC immunotherapy.

**Supplementary Information:**

The online version contains supplementary material available at 10.1186/s12864-024-10243-z.

## Introduction

Globally, the incidence and mortality rates of gastric cancer continue to remain alarmingly high, imposing a significant burden on both society and the economy [1]. The burden imposed by gastric cancer underscores the urgent need for comprehensive strategies and initiatives aimed at prevention, early detection, and effective treatments.

The development of immune-targeted therapy has opened a new chapter in the treatment of gastric cancer. Immunotherapy works by boosting the body’s immune system to effectively attack and destroy cancer cells [2]. However, the development of immunotherapy is still limited by drug resistance and intolerance in the process [3]. It is urgent for us to explore new therapeutic targets in order to overcome these challenges and further advance immunotherapy.

Sphingolipids are a class of lipids with biological activities that play a crucial role in maintaining the structural integrity and functionality of cell membranes [4]. These molecules are composed of a sphingosine backbone, a fatty acid chain, and a polar head group. They are involved in various cellular processes, including cell adhesion, signal transduction, and lipid rafts formation. Recent studies have found that sphingolipid metabolism and downstream signaling pathways are closely associated with the proliferation, metastasis, and immune response of malignant tumors [5]. Researches about precise mechanisms underlying these associations may uncover novel therapeutic targets for gastric cancer therapy.

**Fig. 1:**
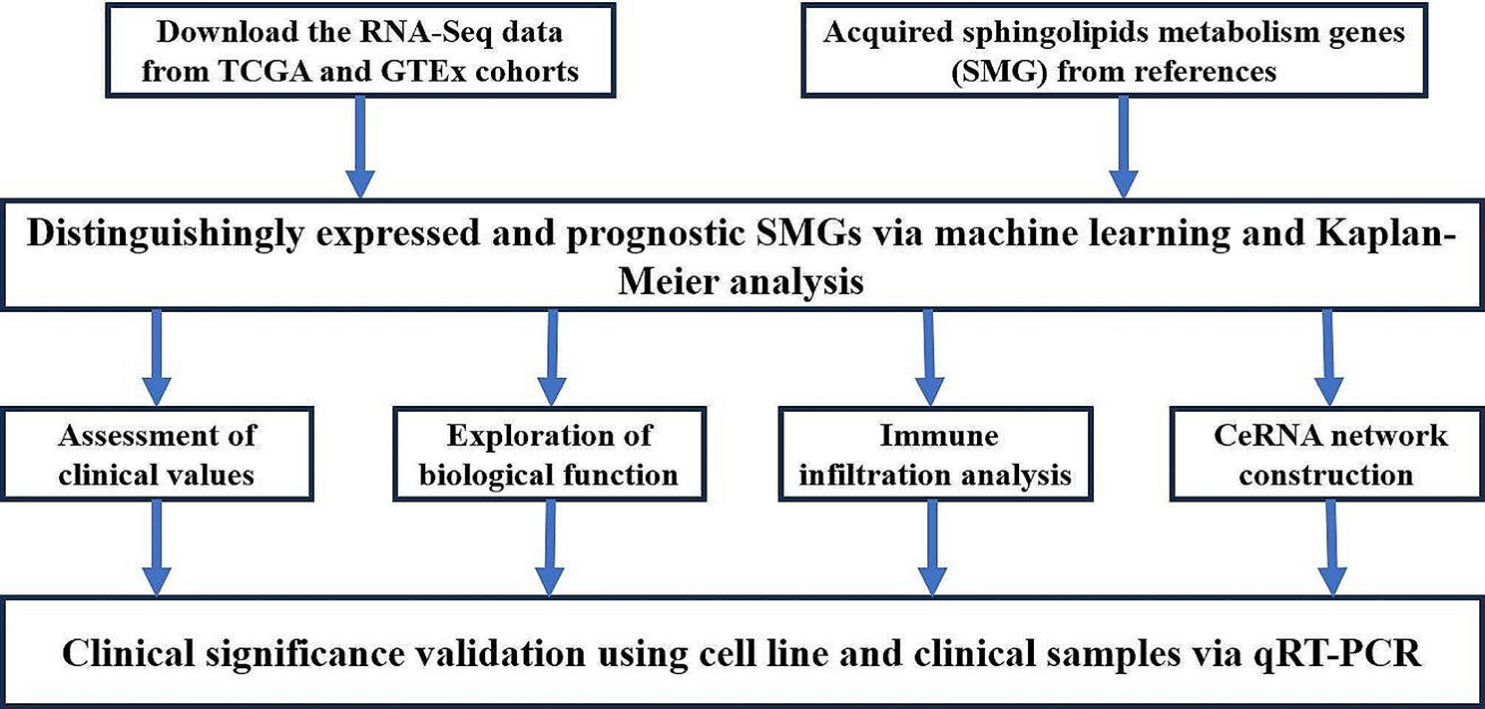
Flow chart of this study

In this study, we aim to utilize machine learning to screen prognostic-related sphingolipid metabolism genes (SMG) to identify novel prognostic and screening biomarkers of sphingolipid metabolism genes in GC for clinical application. We construct a gastric cancer prognosis model from The Cancer Genome Atlas (TCGA) database and normalized RNA-seq data from the Genotype-Tissue Expression (GTEx) data portal as well as clinical samples. Then, we perform multi-omics functional analysis to identify their biological functions and immune infiltration landscape, which can provide new insights for clinical diagnosis, monitoring, and novel adjuvant therapies. The study flow-chart is shown in Fig. 1.

## Materials and methods

### Public database retrieval and clinical samples acquisition

The gene expression profiles and clinical information of gastric cancer patients by The Cancer Genome Atlas (TCGA) database (https://genome-cancer.ucsc.edu/) and normalized RNA-seq data from the Genotype-Tissue Expression (GTEx) data portal (https://www.gtexportal.org/home/index.html). The immortal human stomach cell line GES-1, human GC cell lines (SGC-7903, MGC-803) were purchased from the Shanghai Institute of Biochemistry and Cell Biology, Chinese Academy of Sciences, China. The clinical GC tissues, paired adjacent nontumorous tissues (5 cm away from the edge of GC tissue) were collected from 40 newly diagnostic adult patients with advanced GC received gastrectomy from The First Affiliated Hospital of Ningbo University, China, between 2022 and 2023. All patients participate in the study voluntarily and underwent curative intent resection excluding other malignant tumors. All procedures abide by the Declaration of Helsinki principles and our study is approved by the Ethics Committee of the First Affiliated Hospital of Ningbo University (No. KY2024KY1515).

### Identification of three prognostic-related SMGs via machine learning

Differentially expressed SMGs were identified by Student’s t-test. Least absolute shrinkage and selection operator (LASSO) regression analysis was performed via “glmnet [V 4.1.7]” in R software [V 4.2.1] and tenfold cross-validation was used to determine the penalty regularization parameter λ [6, 7]. Overall survival benefit was assessed by Kaplan–Meier analysis to filter the core genes. Then, univariate regression and multivariate COX regression model was constructed to identify and compute the coefficients of the core genes to build risk score model. Risk score = Coef_SMG1_ * Expression_SMG1_ + Coef_SMG2_ * Expression_SMG2_ + Coef_SMG3_ * Expression_SMG3_ for each patient.

### Construction and validation prognostic nomogram model

As mentioned before, the risk factors in the multivariate regression and risk score model were incorporated into the prognostic model. R package survival [V3.3.1], and rms [V 6.3-0] were performed to build the 1-, 3- and 5-year overall survival time (OS) prediction nomogram model and calibration curves. Concordance index (C-index) was calculated to estimate the discrimination of nomogram. The calibration curves lie on the diagonal 45-degree line suggesting an ideal nomogram model. Decision curve analysis (DCA) curves were generated to evaluate the clinical benefit of our model.

### Evaluation of the diagnostic value of SMGs in GC

Receiver operating characteristic (ROC) curve analysis was performed to assess the diagnostic values of prognostic-related SMGs in TCGA GC cohorts. Combination diagnosis was conducted to improve the diagnostic effect. Clinical samples were used to validate the results.

### Analysis of gene mutation of prognostic-related SMGs

Mutation data was obtained from cBioPortal (https://www.cbioportal.org/), an online tool for cancer genomics [8]. Stomach Adenocarcinoma (TCGA, PanCancer Atlas) was chosen and genetic alternation, cancer subtypes, methylation, copy number alterations (CNAs) were analyzed. MethSurv is a web tool of detecting multivariable survival analysis using DNA methylation data, which was performed to investigate the methylation level of SMGs and prognosis [9].

### Biological function annotation by multi-omics

GeneMANIA prediction server (https://genemania.org/) is an interactive network exploration portal for inferring and visualizing interesting genes [10]. These interactive genes were input into STRING V12.0 (https://cn.string-db.org/) to establish protein-protein interaction (PPI) network [11]. Interactions of high confidence (score ≥ 0.700) were considered.

The competing endogenous RNAs (ceRNAs) hypothesis supposes a reciprocal modulation of ceRNA transcript and of the interacting miRNAs [12]. The miRNAs targeted to the core SMGs and downstream mRNAs were predicted by DIANA-TarBase 8.0 [13]. The long non-coding RNAs (lncRNAs) binding to miRNAs were detected via DIANA-LncBase v3 [14].

KEGG pathway enrichment analysis, gene ontology (GO) classification and Gene Set Enrichment Analysis (GSEA) were used to explore the biological functions and visualized via R packages “clusterProfiler [V 4.4.4]” and “ggplot2”. P value < 0.05 and False Discovery Rates (FDR) < 0.25 represent statistically significant difference.

### Immunity analysis of prognostic-related SMGs

The relationships between the expression level of prognostic-related SMGs and immune cell infiltrations were analysed by R packages “GSVA (1.46.0)” and “estimate (1.0.13)” with the default parameters [15]. Tumor Immunization Single Cell Center (TISCH, http://tisch.comp-genomics.org/home/), a single-cell RNA sequencing database about tumor microenvironment, was performed to investigate the purity and immune infiltration of prognostic-related SMGs in GC [16].

### Establishment of competing endogenous RNA regulatory network

The competing endogenous RNA regulatory network was built on the basis of prognostic-related SMGs. The miRNAs targeted to the prognostic-related SMGs were acquired from TarBase v.9 (https://dianalab.e-ce.uth.gr/tarbasev9) [17]. The modules such as “high experimental throughput”, “direct experimental type”, “high confidence miRNAs only”, “primary interactions only” were selected to refine results with high stringency. Subsequently, the potential associations between miRNAs and mRNAs, long noncoding RNAs (lncRNAs) were explored using starBase (https://rnasysu.com/encori/index.php) [18]. The recommend parameters were set to filter high stringency results: CLIP Data ≥ 3, pan-Cancer ≥ 5. Likewise, RNA binding protein (RBP) interacted with prognostic-related SMGs was screened using the “RBP-Disease”, “RBP-mRNA” modules in starBase as well. CLIP Data ≥ 5 and pan-Cancer ≥ 5 were deemed as high stringency.

### Expression validation by quantitative real-time PCR (qRT-PCR)

The separated tissue was preserved by immediate immersion in RNA save solution (Biological Industries, Israel) in an Eppendorf tube and frozen immediately by immersion in liquid nitrogen for further RNA isolation. All of the RNA was extracted from cells and tissues using TRIzol reagents (Ambion, Carlsbad, CA, USA) based on instructions provided by the kit manual. Total RNA was used as a template and reverse transcribed to cDNA with a GoScript Reverse Transcription (RT) System (Promega, Madison, WI, USA) according to the manufacturer’s instructions [19]. Then, qRT-PCR detection was performed via GoTaq qPCR Master Mix (Promega) whose conditions were as follows: 95°C for 5 min, followed by 40 cycles of 94°C for 15 s, 52°C for 30 s, and 72°C for 30 s. GAPDH mRNA was chosen to normalize and the primer sequences were as follows: GLA: forward, 5’-GCCCCTGAGGTTAATCTTAA-3’, reverse, 5’- AACTGTTCCCGTTGAGACTC-3’; LAMC1: forward, 5’- GCCTTCCTGACCGACTACAACAAC-3’, reverse, 5’- GCGGCTGGTGTGGAACTTGAG-3’; TRAF2: forward, 5’- GATGGGGGTCTTCATCTG-3’, reverse, 5’-CGTAGGTGGATGCCTCC-3’; GAPDH: forward, 5’-ACCCACTCCTCCACCTTTGAC-3’, reverse, 5’-TGTTGCTGTAGCCAAATTCGTT-3’. ΔCt method was used to quantify (ΔCt = Ct_gene_- Ct_GAPDH_). A higher ΔCt value means a lower expression level.

### Statistical analysis

Analyses in this study were flexibly chosen R software (version 4.2.1), cytoscape (version 3.10.1) or GraphPad (version 8.02), and their support packages were as mentioned before. *P* < 0.05 was considered to indicate a significant difference.

## Results

### Construction 3-SMGs signature via LASSO regression analysis

**Fig. 2:**
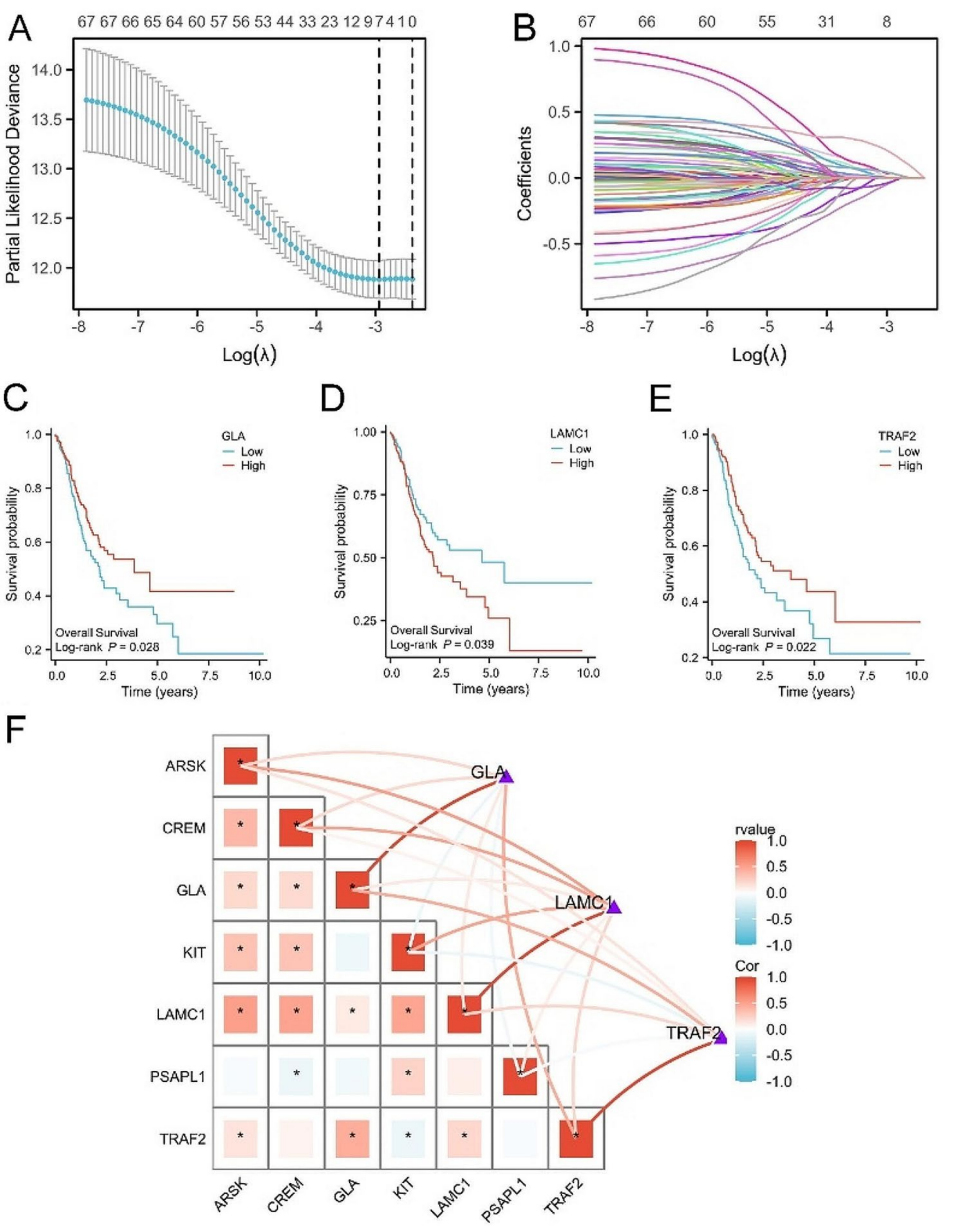
Identification of prognostic related SMGs signature. (**A**) Ten-time cross‐validation for tuning parameter selection in the LASSO model of TCGA GC cohort. (**B**) Coefficient profiles of LASSO model. (**C**-**E**) Kaplan-Meier analysis of the expression of GLA (**C**), LAMC1 (**D**), TRAF2 (**E**) in TCGA GC cohort. (**F**) Co-expression correlation between 3-SMGs signature and prognostic SMGs

97 SMGs were selected (Supplementary Table 1) and 67 SMGs were identified as differentially expressed SMGs by Student’s t-test shown in supplementary Fig. 1 (Fig S1) using TCGA and GTEx samples. For the LASSO regression analysis, 7 candidate genes (ARSK, CREM, GLA, KIT, LAMC1, PSAPL1, TRAF2) were screened from the 67 SMGs in TCGA GC cohorts in Fig. 2A-B. Then, Kaplan–Meier analysis was performed and 3 OS related SMGs were filtered including GLA, LAMC1, TRAF2 (Fig. 2C-E). Co-expression network analysis suggested that the expression level of these SMGs was closely related with risk scores in Fig. 2F.

### Clinical correlation and survival analysis of 3-SMGs signature

**Fig. 3:**
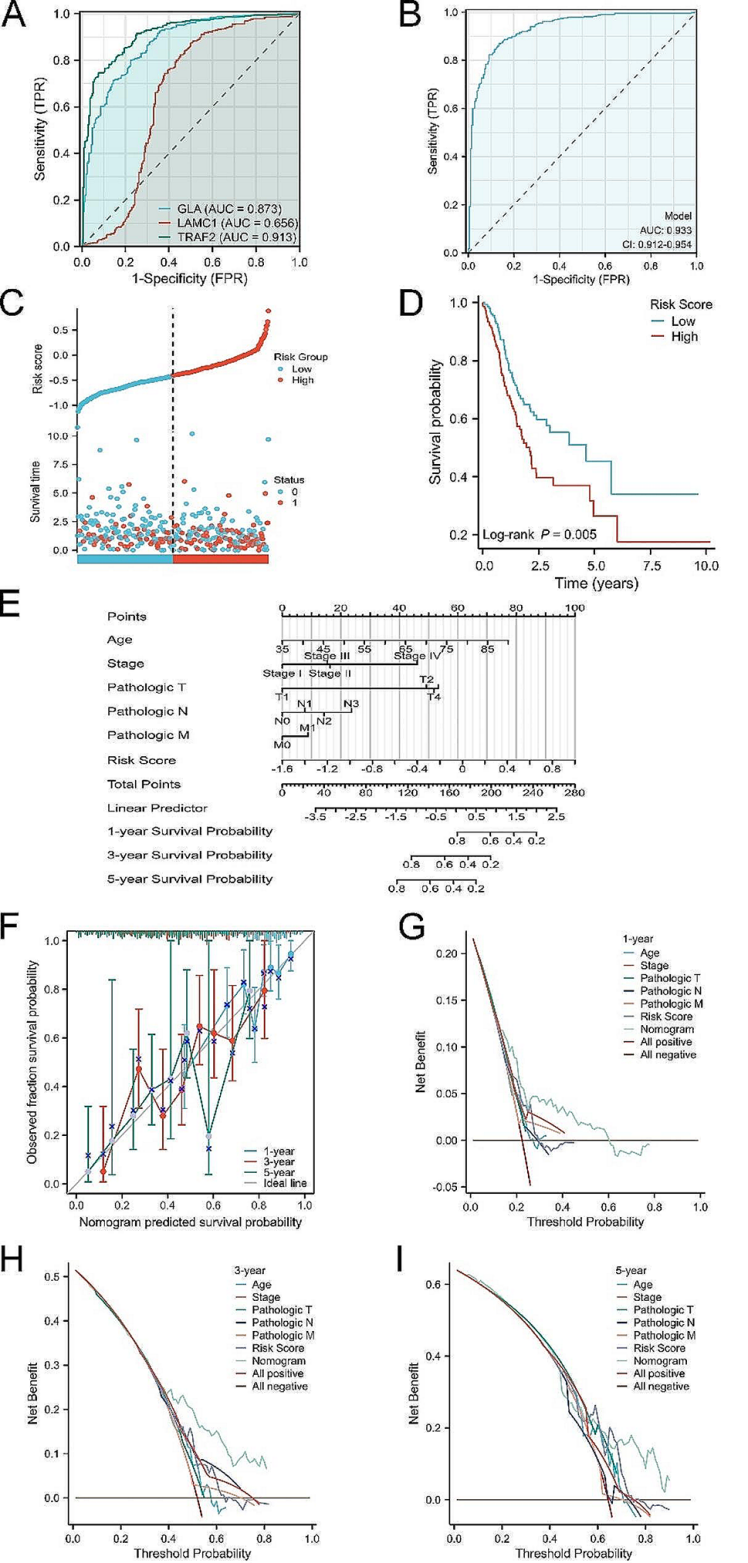
Clinical values explorations of 3-SMGs. (**A**) Diagnostic value of GLA, LAMC1, TRAF2 in TCGA and GTEx cohorts. (**B**) Combing diagnosis of 3-SMGs in TCGA and GTEx cohorts. (**C**) Distribution of risk scores between high- and low- risk groups in TCGA GC cohort. (**D**) Kaplan-Meier analysis of risk model. (**E**) Construction of prognostic nomogram based on risk score. (**F**) The 1, 3, and 5, year calibration plots of the overall survival nomogram model. (**G**-**I**) The 1, 3, and 5, year DCA curves of the nomogram

As the potential significance of 3-SMGs, the clinical values were further dig out. ROC curves were built to calculate the area under the curve (AUC). The results suggested that GLA, LAMC1, TRAF2 can serve as valuable diagnostic markers with clinical application (Fig. 3A-B). Univariate analysis and multivariate analysis Cox regression were performed according to the TCGA GC cohort (Table 1). Based on this result, the risk factor model was established: Risk score = 0.786* GLA exp + 1.296 * LAMC1 exp + 0.771* TRAF2 exp. Subsequently, samples of TCGA GC cohort were divided into high and low group in line with the risk score (Fig. 3C). Kaplan–Meier analysis indicated that higher risk scores accompanied with bad outcome (Fig. 3D, *P* = 0.005). Finally, the prognostic nomogram was constructed shown in Fig. 3E. The C-index of the nomogram model was 0.678 (0.652–0.703), which had a well accuracy illustrated by the calibration curves in Fig. 3F. The 1-year, 3-year, 5-year DCA curves for the risk score model and prognostic nomogram were presented in Fig. 3G-I, implying superior clinical usefulness of the models.

**Table 1:** Univariate analysis and multivariate analysis cox regression in TCGA GC cohort

Characteristics	Total(N)	Univariate analysis		Multivariate analysis	
		Hazard ratio (95% CI)	*P* value	Hazard ratio (95% CI)	*P* value
Age	367	1.022 (1.005–1.039)	**0.009**	1.037 (1.017–1.057)	**< 0.001**
Histologic grade	361				
G1	10	Reference			
G2	134	1.648 (0.400–6.787)	0.489		
G3	217	2.174 (0.535–8.832)	0.278		
Gender	370				
Female	133	Reference			
Male	237	1.267 (0.891–1.804)	0.188		
Stage I	50	Reference		Reference	
Stage II	110	1.551 (0.782–3.078)	0.209	1.529 (0.516–4.528)	0.443
Stage III	149	2.381 (1.256–4.515)	**0.008**	1.487 (0.360–6.139)	0.583
Stage IV	38	3.991 (1.944–8.192)	**< 0.001**	3.317 (0.764–14.410)	0.110
Pathologic T stage	362				
T1	18	Reference		Reference	
T2	78	6.725 (0.913–49.524)	0.061	3.606 (0.451–28.806)	0.226
T3	167	9.548 (1.326–68.748)	**0.025**	4.013 (0.449–35.894)	0.214
T4	99	9.634 (1.323–70.151)	**0.025**	3.851 (0.417–35.570)	0.235
Pathologic N stage	352				
N0	107	Reference		Reference	
N1	97	1.629 (1.001–2.649)	**0.049**	1.220 (0.600–2.480)	0.583
N2	74	1.655 (0.979–2.797)	0.060	1.447 (0.607–3.449)	0.404
N3	74	2.709 (1.669–4.396)	**< 0.001**	1.847 (0.779–4.376)	0.163
Pathologic M stage	352				
M0	327	Reference		Reference	
M1	25	2.254 (1.295–3.924)	**0.004**	1.255 (0.537–2.936)	0.600
GLA	370	0.799 (0.615–1.039)	0.094	0.786 (0.556–1.109)	0.170
LAMC1	370	1.226 (1.047–1.435)	**0.011**	1.296 (1.089–1.543)	**0.004**
TRAF2	370	0.768 (0.590–0.999)	**0.049**	0.771 (0.541–1.098)	0.150

### Biological function exploration of 3-SMGs signature

**Fig. 4:**
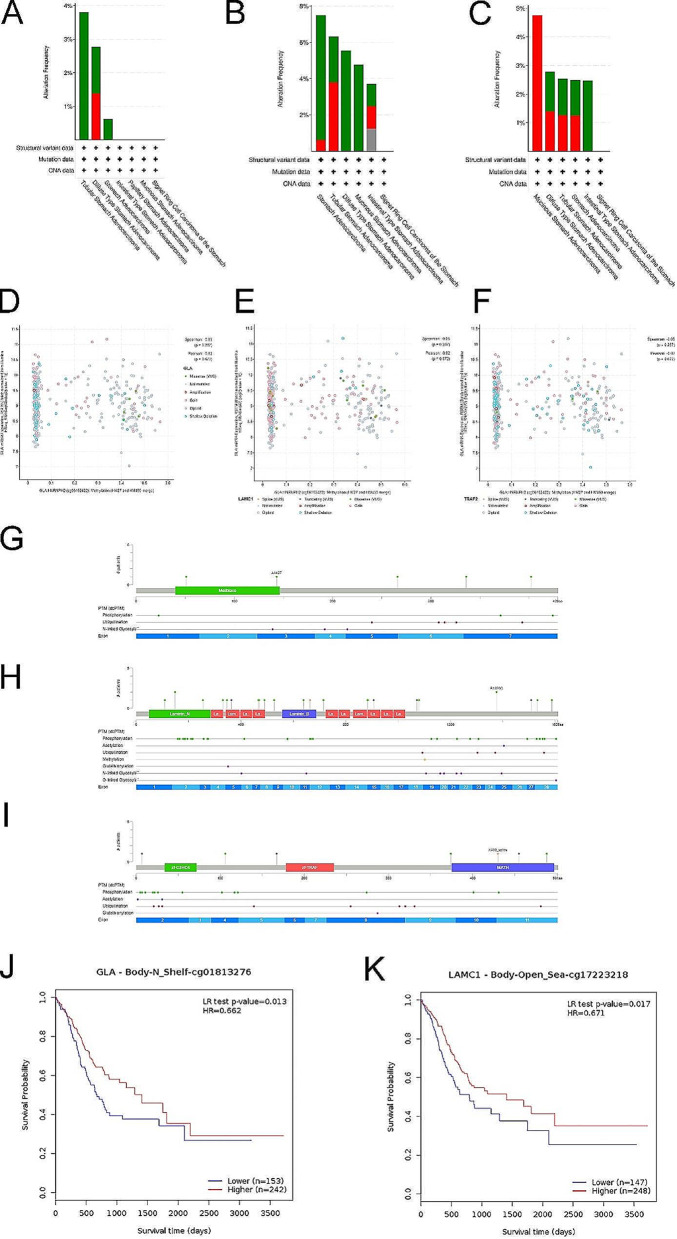
Genomic analysis of 3-SMGs. (**A**-**C**) Alteration frequency, CNA of GLA (**A**), LAMC1 (**B**), TRAF2 (**C**) in GC subtypes. (**D**-**F**) Association between methylation level and alteration of GLA (**D**), LAMC1 (**E**), TRAF2 (**F**) in GC. (**G**-**I**) Phosphorylated mutation sites of GLA, LAMC1, TRAF2. (**J**-**K**) Lower methylation level of GLA, LAMC1 correlated with bad overall survival time of GC

Genomics data from cBioPortal provided a detailed structural description of the cancer genome. The alteration frequency, CNA of GLA, LAMC1, TRAF2 in several subtypes of gastric cancer was revealed in Fig. 4A-C. Likewise, methylation level was also analyzed shown in Fig. 4D-F. Phosphorylated mutation sites of GLA, LAMC1, TRAF2 were displayed in Fig. 4G-I. Moreover, the relationships between methylation level and survival were detected via MethSurv tool, which implied that GLA and LAMC1 methylation associated to GC overall survival time (Fig. 4J-K).

**Fig. 5:**
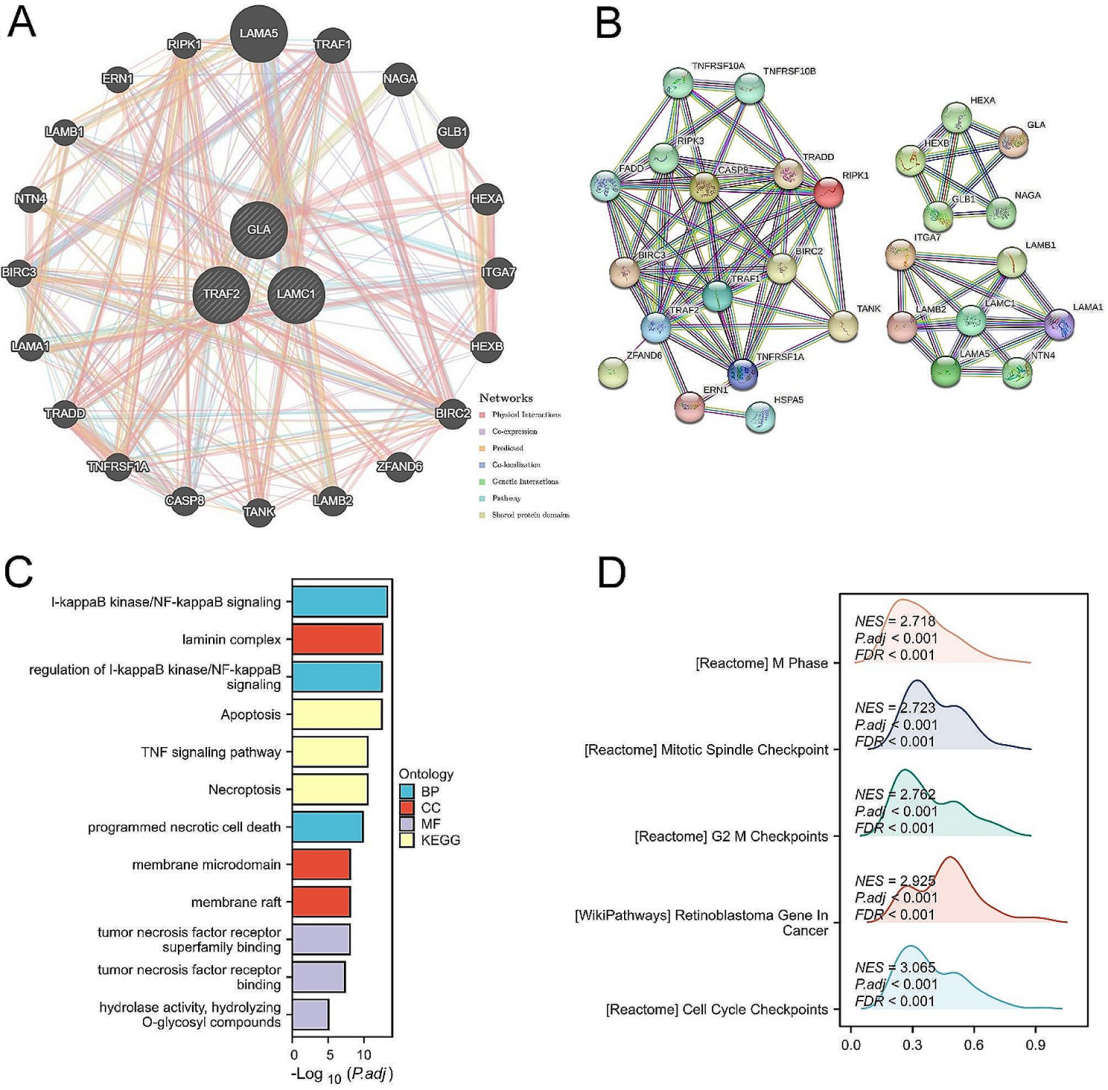
Proteomics analysis and biofunction investigation. (**A**) The top 20 genes associated with 3-SMGs using GeneMANIA. (**B**) Construction of PPI network using top 20 genes. (**C**) KEGG and Go enrichment analysis of PPI network. (**D**) GSEA analysis of PPI network

Then, 3-SMGs was uploaded in GeneMAINA as hub genes and gene interactions network including other 20 genes was built (Fig. 5A). Subsequently, these genes were input into String and constructed PPI network (Fig. 5B). Furthermore, these proteins were used to perform KEGG, Go enrichment analysis, which displayed that the function of these proteins were focused on several signaling pathways and cell death (Fig. 5C). GSEA enrichment analysis disclosed that these proteins influenced cell cycle especially mitotic phase checkpoints (Fig. 5D). In general, 3-SMGs signature may affect cell cycle and death via several signaling pathways to regulate GC progression.

### Immune infiltration landscape of 3-SMGs signature

**Fig. 6:**
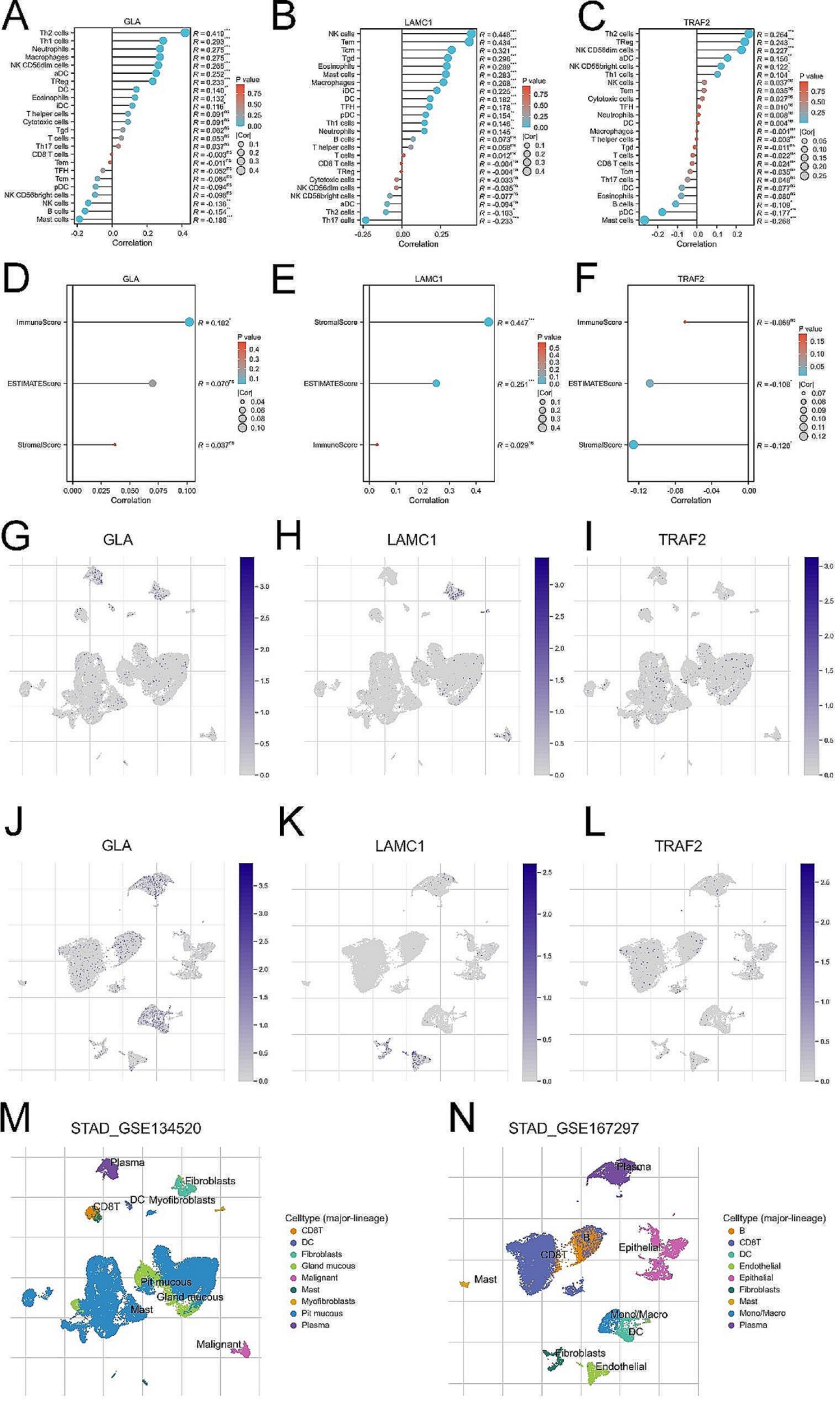
Immune cell infiltration landscape of 3-SMG signature. (**A**-**C**) The lollipop diagram of the expression of GLA, LAMC1, TRAF2 and immune cell infiltration. (**D**-**F**) The stromal score, estimate score, immune score of different expression level of GLA, LAMC1, TRAF2 in TCGA cohort. (**G**-**I**) Annotation of GLA, LAMC1, TRAF2 expression in various cells from STAD_GSE134520. (**J**-**L**) Annotation of GLA, LAMC1, TRAF2 expression in various cells from STAD_GSE167297. (**M**-**N**) Distribution proportion of various immune cells in STAD_GSE134520 and STAD_GSE167297 cohorts

In consideration of altered immune profile may affect tumor progression and patient survival, the relationship between 3-SMG signature and immune cell infiltration in GC microenvironment is essential to investigate [20]. Our results found that the expression of GLA, LAMC1, TRAF2 correlated to several immune cells in TCGA GC cohort (Fig. 6A-C). Meanwhile, the expression of GLA, LAMC1, TRAF2 and immune score, estimate score, stromal score of each sample was estimated displayed in lollipop plot (Fig. 6D-F). Moreover, tumor purity and composition, spatial distribution of immune cells were evaluated via single-cell RNA (scRNA) sequencing by TISCH using GSE134520 and GSE167297 in Fig. 6G-N. All of the results revealed that the set of 3-SMG signature were closely linked with various immune cells infiltration in GC microenvironment.

### Establishment of ceRNA regulatory network

CeRNA is generally considered to form regulatory networks controlling important biological functions and processes in tumorigenesis so the roles of 3-SMGs signature naturally caught our attention [21]. MiRNAs binding to 3-SMGs were retrieved using Tarbase v.9 database and two miRNAs, miR-103a-3p, miR-15b-5p, were co-targeted by GLA, LAMC1, TRAF2. Similarly, the downstream mRNA, lncRNA targets of both miRNAs were screened as well. Totally 25 mRNAs, 4 lncRNAs were selected.

**Fig. 7:**
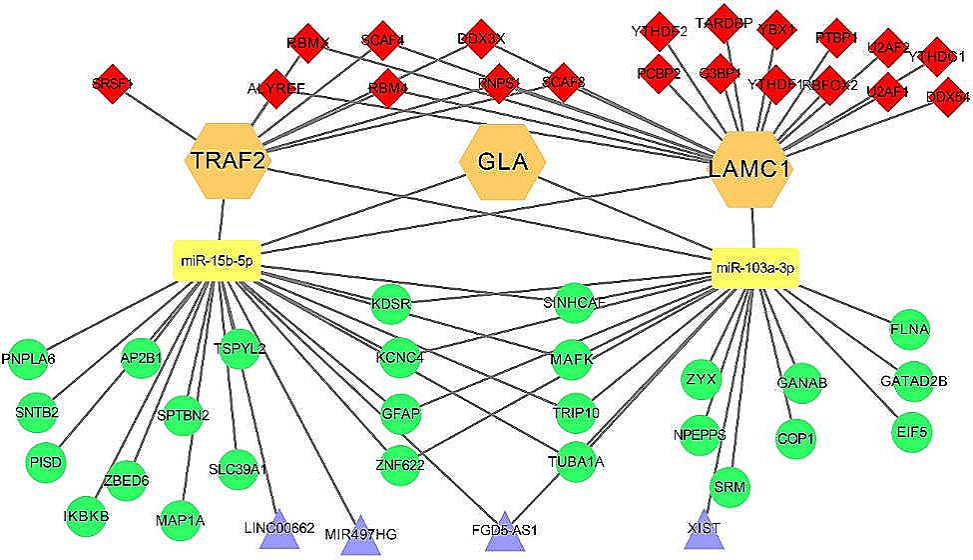
Establishment of RBP-SMGs-miRNAs-lncRNAs/mRNAs regulatory network. Red rhombuses represent RBPs. Orange hexagons represent GLA, LAMC1, TRAF2 mRNAs. Yellow rectangles represent miRNAs. Green circles and purple triangles represent the downstream mRNAs and lncRNAs

As a crucial component of upstream regulator, GC-specific RBPs were investigated and there were 19, 8 eligible RBPs for LAMC1 and TRAF2. Finally, the ceRNA regulatory network was built by cytoscape software in Fig. 7.

### Expression level validation by qRT-PCR

**Fig. 8:**
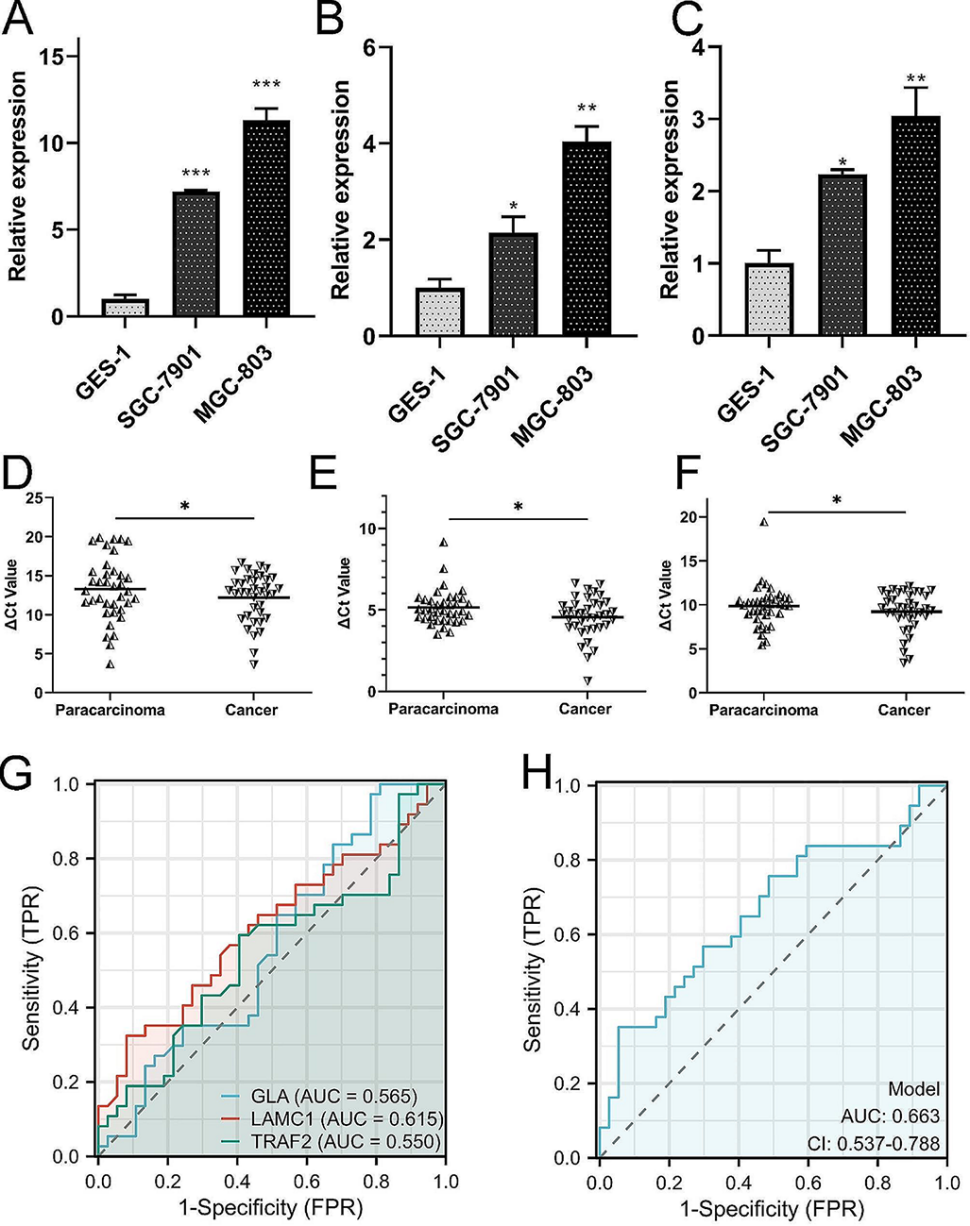
Validation of the differentially expression level and diagnostic values of 3-SMGs. (**A**-**C**) Overexpression GLA (**A**), LAMC1 (**B**), TRAF2 (**C**) in GC cells compared to GES-1. (**D**-**F**) Overexpression GLA (**D**), LAMC1 (**E**), TRAF2 (**F**) in GC tissues compared to paracarcinoma. (**G**) Diagnostic value of GLA, LAMC1, TRAF2 using GC tissues. (**H**) Combing diagnosis of 3-SMGs using GC tissues. (*P < 0.05, ** *P* < 0.01,*** *P* < 0.001)

Cells and clinical samples were used for validating the expression level and clinical significance of 3-SMGs signature. All of 3-SMGs were overexpression in GC cells shown in Fig. 8A-C (*P < 0.05, **P < 0.01, ***P < 0.001). Furthermore, the expression level of 3-SMGs were upregulated in GC tissues compared to paracarcinoma tissues in Fig. 8D-F (*P* < 0.05), which was consistent with the cell line results. The baseline characteristics of the patient was showed in Sup Table 2. Meanwhile, the ROC curves of GLA, LAMC1, TRAF2 were built in Fig. 8G and combined diagnosis was applied to improve diagnosis accuracy in Fig. 8H. All of the results suggested that overexpression 3-SMGs signature were potential GC diagnostic and prognostic biomarkers.

## Discussion

Even to this day, gastric cancer still causes high number of cancer-related deaths worldwide, which is of utmost importance for us to explore novel targets to screen, monitor and targeting therapy [22, 23]. Sphingolipids metabolism is a crucial progress of cell growth, proliferation, apoptosis and emerging evidence point out its roles in meditating tumor initiation, signaling and development [24]. As fewer reports of relationship of sphingolipids metabolism and GC, our study systemically integrates multi-omics data to illustrate the unique characteristics of SMGs in GC, aiming at providing theoretical supports and clinical application targets.

In this study, 67 SMGs were firstly identified as differentially expressed SMGs. Then, LASSO regression analysis was performed to filter 3 prognostic related SMGs including GLA, LAMC1, TRAF2. GLA is a vital gene regulating glycosphingolipid metabolism and mutation of GLA can cause glycosphingolipid accumulation and life-threatening, multi-organ complications [25]. The influence of overexpression GLA in malignant tumors even in GC remains unknown. Recent researches have proven LAMC1 is a critical prognostic factor and potential target in several tumors, which is a promising target for future therapy [26, 27]. It has been convinced that TRAF2 promote M2-polarized tumor-related macrophage infiltration, cancer progression and angiogenesis and by decreasing autophagy in clear cell renal cell carcinoma [28]. All evidence also indicated the importance of 3-SMGs and the significance of them is worthy to dig out. Our PCR data further proved these overexpression level and diagnosed value. Going further, we evaluated prognostic values and constructed risk model and novel prognostic nomogram according to TCGA cohort, which is worthy for validating in future, prospective, multi-center clinical trials.

Then, multi-omics data was used to examine the biological functions of 3-SMGs signature. Genomic data reveals the alteration frequency, CNA and methylation level of 3-SMGs. As mentioned before, the mutation of GLA can trigger decreased/absent α-galactosidase activity even fabry disease. Meanwhile, enzyme activity and substrate/byproduct accumulation are important in tumor diagnosis and disease-monitoring biomarkers, which implies the future application of GLA [29]. It has been proven that LAMC1 rarely methylated in breast cancer but our result illustrated the role of LAMC1 methylation in GC outcome, which suggests the prognostic significance of LAMC1 as well [30]. Subsequently, gene, proteins interact networks were built and biological functions were analyzed. Our results showed that 3-SMGs signature correlated to cell cycle and death via several signaling pathways to GC initiation and progression, which provides directions for further molecular biological research.

As targeted and immune therapies receive more and more attentions, it is pivotal to understand the immune landscape of tumor microenvironment and host immunity for combining chemotherapy with immunotherapy [31]. Spitzel M found that expression of GLA correlated to dysregulation of immune response in fabry disease, prompting its role in immunotherapy response [32]. Similarly, recent research pointed out that overexpression LAMC1 took part in the immune response and immune infiltration in diabetic kidney disease according to the immunohistochemistry results, which indicates the unique role of LAMC1 in the immune therapy in the future [33]. Wu’s study revealed LILRB3 regulated T-cell antitumor immune responses through the TRAF2-cFLIP-NF-κB signaling axis, which can be reversed via blocking TRAF2 signaling with antagonizing antibodies [34]. In this study, the association between 3-SMGs signature and GC microenvironment was further deeply excavated, offering new insights into the future GC immunity and how this information can be harnessed towards effective personalized immunotherapy strategies.

Increasing investigations of miRNA regulatory mechanisms in tumors have been greatly expanded by recent findings of ceRNA network, which is the main mechanism of mRNAs and lncRNAs in human cancers [35]. At the same time, given the role of RBPs in cancer emerges, the synergic or competitive ability of RBPs to interact with various downstream RNAs make it an appropriate group to be selectively dysregulated in cancer [36]. Our research provided an innovatively prospect about integrating both regulatory mechanisms and built a novel RBP-SMGs-miRNA-mRNAs/lncRNAs regulatory network. Interestingly, the co-target such as lncRNA FGD5-S1, ZNF622 may be important targets for future studies and GC therapy.

There are still some limitations in our study. Truly, the result of the diagnostic AUC in validation cohort is consistent with the public data but it is not as high as the test cohort in TCGA and GTEx. We suppose the fold changes may possibly be the minor discrepancy between the qRT-PCR expression levels and high-throughput sequencing themselves in the sensitivity and specificity [37–38]. Nevertheless, the diagnostic efficiency of 3-SMGs signature is much better than traditional tumor biomarkers such as carcinoembryonic antigen (CEA), carbohydrate antigens 19 − 9 (CA19-9), CA125 [39]. Thus, we believe this 3-SMGs signature still has promising future with a low-cost by PCR. Likewise, though our nomogram has an amazing AUC in prognosis, clinical monitor even treatment decisions should be guided by both nomogram and entry criteria especially in tumor recrudesce patients rather than merely nomogram estimated risk [40]. In summary, large randomized clinical trials are essential to confirm the superiority of our model.

In conclusion, upregulated 3-SMGs signature are excellent predictive diagnosed and prognostic biomarkers, providing a new perspective for future GC immunotherapy.

### Electronic supplementary material

Below is the link to the electronic supplementary material.

Supplementary Material 1

Supplementary Material 2

## Data Availability

Original datasets are available in a publicly accessible repository: The original contributions presented in the study are publicly available. These data can be found online UCSC XENA [https://xenabrowser.net/datapages/], including: TCGA [https://xenabrowser.net/datapages/?dataset=tcga_RSEM_gene_tpm&host=https%3A%2F%2Ftoil.xenahubs.net&removeHub=https%3A%2F%2Fxena.treehouse.gi.ucsc.edu%3A443], GTEx [https://xenabrowser.net/datapages/?dataset=gtex_RSEM_gene_tpm&host=https%3A%2F%2Ftoil.xenahubs.net&removeHub=https%3A%2F%2Fxena.treehouse.gi.ucsc.edu%3A443]. Further inquiries can be directed to the corresponding authors.
