# Supplementary material for: Machine learning to establish three sphingolipid metabolism genes signature to characterize the immune landscape and prognosis of patients with gastric cancer
Source: BMC Genomics. 2024 Mar 28;25:319. doi: 10.1186/s12864-024-10243-z (PMC10976768; doi:10.1186/s12864-024-10243-z)
Supplement: Supplementary file 1 — Supplementary Material 1 [file 12864_2024_10243_MOESM1_ESM.docx]

Supplementary Material

## Supplementary Table

**Supplementary Table 1 97 Sphingolipid metabolism genes**

| **Sphingolipid metabolism genes** |
| --- |
| A4GALT |
| ACER1 |
| ACER2 |
| ACER3 |
| ALOX12B |
| ALOXE3 |
| ARSA |
| ARSB |
| ARSD |
| ARSF |
| ARSG |
| ARSH |
| ARSI |
| ARSJ |
| ARSK |
| ARV1 |
| ASAH1 |
| ASAH2 |
| B4GALNT1 |
| BAX |
| CERK |
| CERS1 |
| CERS2 |
| CERS3 |
| CERS4 |
| CERS5 |
| CERS6 |
| CREM |
| CSNK1G2 |
| CTSA |
| DEGS1 |
| DEGS2 |
| ELOVL1 |
| ENPP7 |
| FA2H |
| GAL3ST1 |
| GALC |
| GBA |
| GBA2 |
| GLA |
| GLB1 |
| GM2A |
| HEXA |
| HEXB |
| IL2 |
| KDSR |
| KIT |
| LAMA1 |
| LAMB1 |
| LAMC1 |
| LYN |
| MAP7 |
| NEU1 |
| NEU3 |
| NEU4 |
| PPM1L |
| PPT1 |
| PRKD1 |
| PSAP |
| PSAPL1 |
| S1PR1 |
| SELL |
| SELP |
| SERINC1 |
| SFTPB |
| SGMS1 |
| SGMS2 |
| SGPL1 |
| SGPP1 |
| SGPP2 |
| SMPD1 |
| SMPD2 |
| SMPD3 |
| SMPD4 |
| SPHK1 |
| SPHK2 |
| SPNS2 |
| SPTLC1 |
| SPTLC2 |
| SPTLC3 |
| SPTSSA |
| SPTSSB |
| ST3GAL5 |
| ST6GALNAC3 |
| ST6GALNAC5 |
| ST6GALNAC6 |
| ST8SIA1 |
| ST8SIA3 |
| ST8SIA5 |
| STS |
| SUMF1 |
| TEX2 |
| TH |
| TRAF2 |
| UGCG |
| VAPA |
| VAPB |

| **Supplementary Table 2 Pathologic characteristics of clinical GC samples** | |
| --- | --- |
| Characteristic | n (%) |
| Age |  |
| <=65 | 23 (57.5%) |
| >65 | 17 (42.5%) |
| Gender |  |
| Female | 9 (22.5%) |
| Male | 31 (77.5%) |
| T stage |  |
| T1 | 0 |
| T2 | 3 (7.5%) |
| T3 | 10 (25.0%) |
| T4 | 27 (67.5%) |
| N stage |  |
| N0 | 7 (17.5%) |
| N1 | 7 (17.5%) |
| N2 | 7 (17.5%) |
| N3 | 9 (22.5%) |
| M stage |  |
| M0 | 32 (80.0%) |
| M1 | 8 (20.0%) |
| Pathologic stage |  |
| Stage I | 0 |
| Stage II | 7 (17.5%) |
| Stage III | 25 (62.5%) |
| Stage IV | 8 (20.0%) |
| Perineural invasion |  |
| No | 17 (42.5%) |
| Yes | 23 (57.5%) |
| Vascular invasion |  |
| No | 13 (32.5%) |
| Yes | 27 (67.5%) |


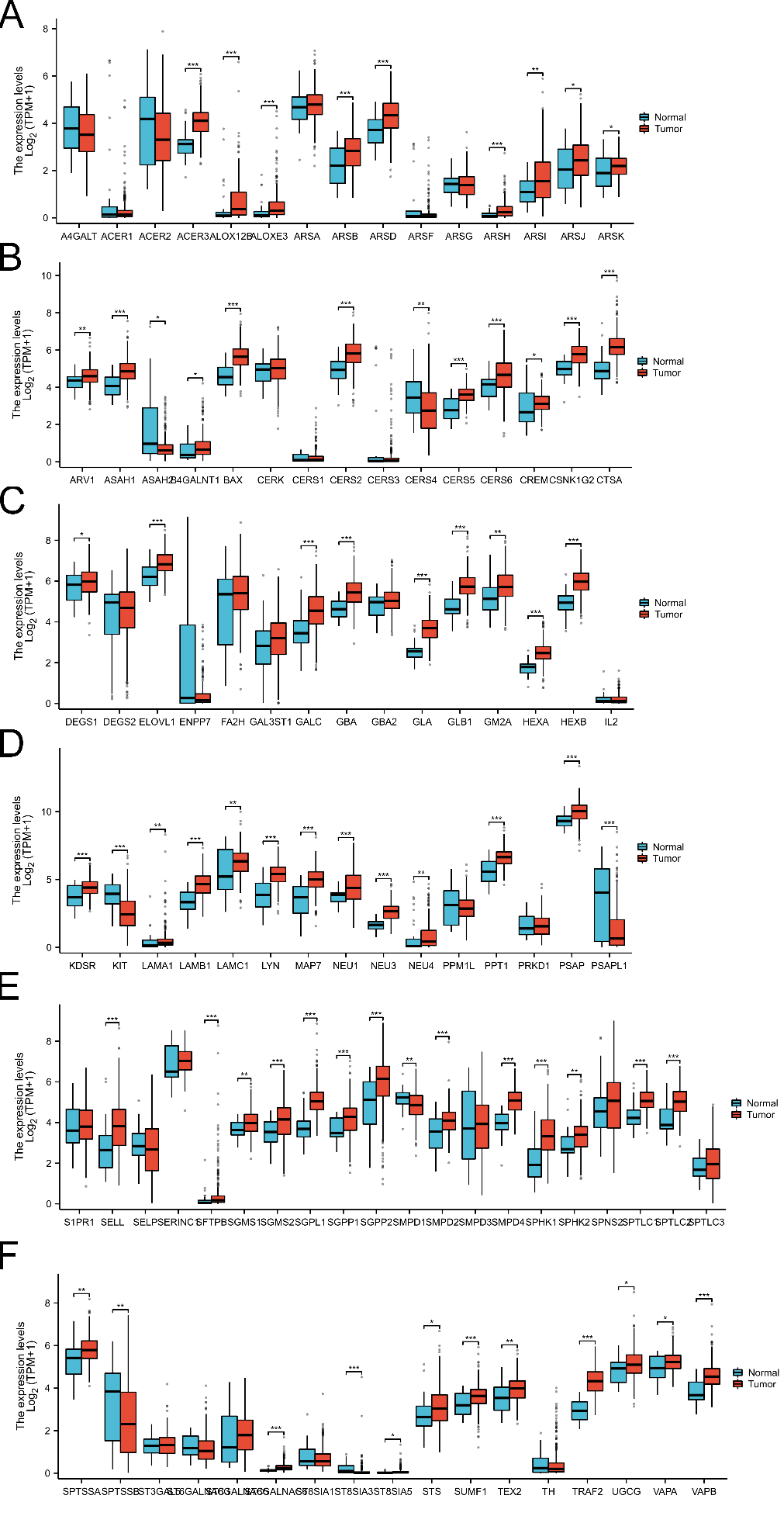
**Supplementary Figure 1.** (A-F) Expression level of 67 SMGs using TCGA and GTEx samples (*P<0.05, ** P<0.01, *** P<0.001).
