# Supplementary material for: Machine learning to establish three sphingolipid metabolism genes signature to characterize the immune landscape and prognosis of patients with gastric cancer
Source: BMC Genomics. 2024 Mar 28;25:319. doi: 10.1186/s12864-024-10243-z (PMC10976768; doi:10.1186/s12864-024-10243-z)
Supplement: Supplementary file 2 — Supplementary Material 2 [file 12864_2024_10243_MOESM2_ESM.pdf]

Checklist for initial submissions

Your manuscript will be subject to a quality check before it can be presented to an Editorial Board Member (EBM) for assessment. This is to ensure that all of the information required by the EBM and peer-reviewers is available.

Please review the checklist before submitting, as manuscripts that do not comply may be returned for changes before they are assigned to an EBM. Additionally, please ensure that all author information entered both in your manuscript and on our system is accurate.

Should you have any questions regarding this checklist, please contact us at srep@nature.com

**Submission Files**			
**Item**	**Permissible file format**	**Notes**	**Completed**
Cover letter	.doc, .docx, .pdf	Will be seen by the EBM but not the peer-reviewers	☑
Manuscript File	.doc, .docx, .tex, .pdf	Only one file permitted	☑
Main Figure File(s)	.jpg, .eps, .tiff, .psd, .png, .pdf	Can be uploaded separately or included within the Manuscript file	☑
Main Table(s)	Do not upload separately	Must be included in the Manuscript File	☑
Supplementary Information	.txt, .gif, .html, .doc, .jpg, .swf, .mov, .xlsx, .pdf, .ppt, .wav, .csv, .zip	Must be included separately from the Manuscript File, preferably as a single file. Supplementary datasets should be provided as separate .xlsx files	☑

**Manuscript File**	**Completed**
**TITLE**	
Exactly matches the title entered on the submission system and in any supplementary information	☑
The title should be a precise statement describing the main message of the paper and not contain puns or idiomatic expressions	☑
**AUTHORS**	
Corresponding author(s) are identified using an asterisk. Email address(es) of corresponding author(s) should be provided on the title page	☑

All author (corresponding and contributing) details are included on the submission system as well as in the Manuscript File	☑
Affiliations (including country) are provided and linked to authors with superscript numbers	☑
For papers with consortia as part of the authorship, please include the consortium name as a main author in the author list on the title page. Please provide a full list of members of this consortium at the end of your Manuscript File, after the references, providing affiliations marked by superscript numbers as per the main author list. The consortium name should also be entered as an author on the submission system, together with the contact details of a nominated consortia representative	☑
**ABSTRACT**	
Exactly matches the abstract entered on the submission system	☑

Does not contain citations	☑
Does not include subheadings or inline subheadings (i.e. not a structured abstract)	☑
**MAIN TEXT**	
No footnotes	☑
Tracked changes must not be used in the Manuscript File. If you wish to highlight any changes made in your Manuscript File, please do so with the highlighter tool or by changing the colour of the font. Please do not highlight changes in the text by italicising. You may upload a version containing tracked changes as a 'Related Manuscript' file, as long as a clean version of the Manuscript File is also provided	☑
**METHODS**	
Included in Manuscript File and contain sufficient detail to repeat experiments	☑
For experiments involving live vertebrates and/or higher invertebrates, the Methods section must include a statement that:<br>1. Identifies the institutional and/or licensing committee that approved the experiments, including any relevant details.<br>2. Confirms that all experiments were performed in accordance with relevant named guidelines and regulations.<br>3. Confirms that the authors complied with the ARRIVE guidelines.	☑
For experiments involving human subjects (or tissue samples) the Methods section must include a statement that:<br>1. Identifies the institutional and/or licensing committee that approved the experiments, including any relevant details.<br>2. Confirms that all experiments were performed in accordance with relevant named guidelines and regulations.<br>3. Confirms that informed consent was obtained from all participants and/or their legal guardians.	☑

Please note that:<br>1. Study participant names (and other personally identifiable information) must be removed from all text/figures/tables/images.<br>2. For manuscripts that include information or images that could lead to the identification of a study participant, your Methods section must include a statement that confirms informed consent was obtained to publish the information/image(s) in an online open access publication. The use of coloured bars/shapes or blurring to obscure the eyes/facial region of study participants is not an acceptable means of anonymisation.	☑
Scientific Reports will not consider manuscripts describing research that involves organs/tissues procured from prisoners. In addition to the above requirements for experiments involving human subjects (or tissue samples) authors of manuscripts describing human transplantation research must:<br>1. Include a statement in their manuscript attesting that no organs/tissues were procured from prisoners<br>2. Name all institution(s)/clinic(s)/department(s) via which organs/tissues were procured<br>3. Confirm that informed consent was obtained from both organ donor(s) and recipient(s)	☑
**AUTHOR CONTRIBUTIONS STATEMENT**	
Author contributions statement specifying the individual contribution of each author, is provided. This should be included after the references, and every author's contribution must be listed	☑
**ADDITIONAL INFORMATION**	
Competing interests statement is provided in the Manuscript File under the heading "Additional Information", and matches the information entered on the submission system. Competing financial and non-financial interests should be disclosed	☑
**LEGENDS**	
A legend is provided for each main table and main figure in the Manuscript File. These should be placed at the end of the manuscript, after the references	☑

For supplementary figures and tables, include a brief title and legend (incorporated into the file to appear near the image)	☑
Only legends for main display items should be included in the Manuscript File; legends for supplementary figures and tables should be included in the Supplementary Information file	☑
**DATA AVAILABILITY STATEMENT**	
A Data Availability Statement is provided as a separate section (titled 'Data Availability') at the end of the main text, before the 'References' section. See the 'Availability of materials and data' policy for statement requirements	☑
**REFERENCES**	
In a numbered list and all in-line citations should be matched back to a reference via numbering within square brackets	☑

**Display Items**	
**MAIN FIGURES**	
Schemes, graphical abstracts and boxes are not permitted and must be labelled/numbered as either a figure or table as appropriate. As an online journal, we are unable to accept any 'Cover' images or table of contents figures	☐ ✓
Gel and blot images are presented in compliance with our digital image and integrity policies. Where cropped gels/blots are displayed, this should be noted in the figure legend; full-length/uncropped gels and blots should be included in a Supplementary Information file	☐ ✓
**MAIN TABLES**	
Main tables are provided in the Manuscript File, and not as separate files, in an editable format (not embedded as an image in the document)	☐ ✓
**Supplementary Information**	
Supplementary material is uploaded separately, and not included in the Manuscript File	☐ ✓
